# Resignation of Prof. Mott

**Published:** 1850-08

**Authors:** 


From the N. Y. Medical Gazette we glean the following items of medical news:

RESIGNATION OF PROF. MOTT.

Dr. Valentine Mott, professor of surgery in the University of New York, has tendered to the proper officers his resignation. The cause which led him to take this step, was the appointment of Dr. Detmold to the chair of Theory and Practice. Dr. Mott, it appears, was in Europe at the time, and transmitted his resignation, conditioned upon the continuance of the appointment of Dr. Detmold; whereupon Dr. Detmold promptly resigned the chair to which he had been appointed, and it is understood *both* resignations have been accepted.

